# Report on Vegetable Parasites

**Published:** 1870-02

**Authors:** N. S. Davis

**Affiliations:** Chairman Committee


ARTICLE VI.

### REPORT ON VEGETABLE PARASITES.

PRESENTED TO THE ILLINOIS STATE MICROSCOPICAL SOCIETY, JAN. 14, 1870.

By N. S. DAVIS, M.D., Chairman Committee.

[Published by Request of the Society.]

Parasites are living organizations that attach themselves to, and derive their nourishment from, some other organic body. In one sense, vegetable parasites are such vegetations as attach to, and derive their nourishment from, other vegetable growths. In another sense, they embrace all parasitic growths of vegetable organization, whether attaching to other bodies of vegetable or of animal nature; and it is in this sense that we will use the term vegetable parasite, in this report.

We may divide the whole into two groups or classes. The first embraces such as are found attached to living vegetables only. The second, such as may spring up on either vegetable or animal matter, whether living or dead, under certain combinations of heat, moisture, and light. The first of these classes may be conveniently divided into two orders, namely: the green parasites, or such as have leaves or organs of respiration, through which they imbibe a part of their nourishment from the atmosphere; and the brown, which have no leaves or respiratory organs, but derive all their nourishment from the roots of the vegetables to which they are attached. They originate under the surface of the ground and shun the light. The first of these orders are described chiefly under the names of *Viscum* and *Loranthus*, while the second are represented by the *Orabanche* and the *Latharie*.

The second class are termed *Fungi* and *Entophyta;* or, in more familiar language, as mushrooms, mould, mildew, smut, rust, blight, etc. They belong to the class *Cryptogamia* of Linneus, and are distinguished from some of the other orders of that class, chiefly by their deriving their nourishment solely from the organic matter to which they are attached.

They are very numerous, Berkely having given about 600 *genera* and 4000 species, to which other investigators have added many more since.

To the unassisted eye, few things appear more dissimilar than the large fungous excressences on forest trees, and the *mucedo*, or filiment of mould; yet, under the microscope, all the varieties of fungi appear to possess a similar organization. They consist primarily of simple cells or spores, united in the form of lines. These may exist separately in the form of extremely delicate filaments, as the *mucor*, or common mould, or they may be aggregrated in large masses of considerable density, as in the well-known *boleti*, on the forest trees. Any detailed account of these primary formations, or attempt at classification, would be more tedious than profitable to this Society. For our present purpose, we may divide the whole of what we have denominated the second class into two groups. The first embraces such as are found growing on vegetable products, either living or dead, and constitute the Fungi proper; the second embraces such as are found attached to animal structures, more especially such as are covered with epithelial cells, like the skin and mucous membranes, these have been termed *Entophyta*.

Many of those embraced in the first group are of great importance to the agriculturist and fruit grower, because they are found to attack, and greatly injure, in different seasons, almost every variety of grain, fruit-bearing vegetables, and trees. The rust, smut, and mildew of wheat, rye, and corn, and the blight of the plum, cherry, and pear trees are familiar examples, and are worthy of the most patient and careful study.

Many of the orders and species embraced in the second group, attaching themselves to man and animals, and found connected with various diseased conditions, have more particularly attracted the attention of members of the profession to which I belong. For many years it has been claimed by some of the more eminent men in the medical profession, that some of the most common and important diseases that afflict man, both endemic and epidemic, are produced by vegetable parasites, belonging either to the class *algœ* or *fungi.* Nearly 30 years since, Dr. J. K. Mitchell, then Professor of Practical Medicine, in the Jefferson Medical College, in Philadelphia, published a small work, embodying facts and observations strongly calculated to induce the conviction that our well-known intermittent and remittent fevers were produced by a species of microscopic fungi, developed on decaying vegetable matter, under the influence of heat and moisture. In 1855, M. Robin, of Paris, published his work called "Natural History of the Parasitic Vegetables, which Grow on Man and Living Animals." In this work the author enumerates ten species that attack the skin, and twelve the mucous membranes. As most of these are mere varieties, other writers have reduced those attacking the skin to three, namely: the *Tricophyton*, *Microsporon* and *Achorion Schœnlenii.* They present the most simple form of vegetable organization, consisting of mere spores, with occasional additions of *Mycelium.* They have been found in the different varieties of *Favus* or *Porrigo*, one variety of *Pityriasis*, *Mentagra*, and some forms of *Herpes* and *Eczema.* Thus far they have been found a constant accompaniment of such diseases only, as attack the hair bulbs, and follicles of the skin. Whether these parasites are the specific cause of the cutaneous diseases in connection with which they are found, or only accompaniments, is not fully determined. Most of the French and German investigators advocate the affirmative, while others, especially in England and this country, claim the negative. Erasmus Wilson, for instance, contends that the so-called parasites are properly degenerated products of epithelial and other cell formations, and are consequences instead of causes of the cutaneous diseases. Notwithstanding the recent investigations of Koebner, Pick, and others, we think the view taken by Wilson is more nearly in accordance with all the facts.

Of the twelve varieties of vegetable parasites enumerated by Robin as attacking the mucous membrane, six are varieties of *Leptonitus*, one of *Torula*, one of *Merismopœdia*, one *Oscillaria*, and one *Oidium.* To these may be added the *Cholera Fungus*, of Brittan and Budd, and the *Palmellœ* or Malaria Fungus of Saulsbury.

Although the varieties of parasitic growths mentioned by Robin have been observed on all parts of the mucous membranes of the digestive and urinary organs, only the *oidium* has been identified as uniformly connected with particular diseases.

This latter constitutes a large part of the white curdy exudation on the mucous membrane of the mouth, in thrush or miguet, and in diphtheria. The branch of our subject which has attracted most attention during the last few years, is that relating to the dependence of epidemic cholera on a parasitic growth, belonging to the order *Torulœ* or *Oidium.*

Brittan, Budd, Hillier, and others claim to have established the fact that the characteristic cholera evacuations by vomiting and purging, contain large numbers of vegetable spores, capable of rapid reproduction or multiplication. As seen in the recent rice-water discharges, they appear like the simplest form of *Sporule* or *Torula*, but by cultivation they are found to develope into the form of *Oidiœ.* It has been claimed and extensively credited, that these minute organizations were the active cause of cholera, and that the disease was actively propagated in communities, and from one place to another, by the cholera dejections containing these spores. During the last cholera epidemic in this city, 1866, we undertook a series of microscopic examinations, for the purpose of satisfying ourselves concerning a subject of so much importance.

We procured, in clean vials, samples of the pure rice-water discharges, of several patients in the active stage of cholera. Some of these were examined with a high magnifying power, within one hour after they were voided, and the examination was repeated daily, for five or six days. Pencil sketches were made of several of the views presented. In all the earlier examinations, numerous minute, round, or oval bodies appeared in the field of the microscope, resembling the primary sporules of the *Torula*, but none united together in linear form. After standing, however, from two to four days, these were, in a few instances, apparently united in the form of *Oidium;* and in one that was examined, after standing six days, there appeared two specimens resembling the penicillium.

[The speaker here drew an illustration on the blackboard, showing the field of the microscope.]

In addition to these vegetable parasites, all the specimens examined by me presented large numbers of *vibriones*, and two specimens of animalcule, differing from any that I had seen before. So far, the results of my microscopic investigations only corroborated the results of similar examinations made by others. But the question then came up in my mind, whether these parasitic formations were peculiar to the discharges of epidemic cholera, or were also to be found in any serous evacuation from the alimentary canal, such as occur in ordinary cholera morbus, cholera infantum, and serous diarrhœa. To determine this question, since the cholera epidemic has disappeared, we have from time to time procured and examined samples of the discharges in the last named diseases; and in no instance have we failed to find more or less of minute spores or bodies, which appear to me identical with those found in the discharges of the patients laboring under epidemic cholera. We are, therefore, by no means prepared to consider the origin and propagation of epidemic cholera as settled.

The subject requires much more extended investigation before we can draw reliable conclusions.

It has long been known that any organic substance containing *proteine*, even in small quantity, in a moist state, exposed to a temperature of 70 to 80 degrees, with access to oxygen, presents, in a few days, products which under a good magnifying power have all the characteristics of vegetable spores.

If further observations should confirm the fact, that the fungous bodies described by Brittan, Hillier, and others, were present in the serous discharges from the alimentary canal, in other diseases beside epidemic cholera, it would not only disprove the theory that the last-named disease was produced by such fungi, but it would go far to establish the position that they were the products of morbid secretions, instead of the cause. It is a fact worthy of note, that up to the present time, vegetable parasites have been found only in connection with diseases of such structures as are covered with epethelial cells, like the skin and mucous membrane, or on suppurative surfaces, where the organic cells are undergoing more or less degeneration. This legitimately suggests the question whether all these parasitic forms of matter are not the results of what Erasmus Wilson has styled *phytiform*—degeneration of cells and granules of animal matter? But aside from all such questions of a histological character, the study of parasitic growths in connection with the prevalence of diseases, especially of an epidemic character, is of the highest importance in its bearing on sanitary regulations and the health of communities. In this direction, some things of great importance may be considered as already settled. Whatever may be the differences of opinion among observers, in relation to the nature and mode of development of these simple parasitic bodies, found in connection with epidemic and some endemic diseases, all agree that their production and rapid multiplication are directly dependent on the coëxistence of three things, *viz.:* organic matter capable of undergoing degeneration or decay, moisture, and a temperature generally above 60 degress F. If, with these, we have coincidently the exclusion of sunlight, we have the conditions most favorable for all vegetable parasitic formations. If the coöperation of these causes will generate a cholera fungus in the Valley of the

Ganges, we see no reason why they will not do the same in the Valley of the Mississippi, or in Chicago. But whether the cholera fungus is generated here or imported from the other hemisphere, it is certain that without the coöperation of the three first-named conditions, neither it or any other fungus can be propagated, to the injury of individual or public health. Hence, whatever contributes to the prompt removal, from this or any other city, of the decomposable organic mattter, the surplus water or moisture, and gives to all its inhabitants air and sunlight, contribute directly to the preservation of public health.

